# Contaminated Ventilator Air Flow Sensor Linked to *Bacillus cereus* Colonization of Newborns 

**DOI:** 10.3201/eid1905.12039

**Published:** 2013-05

**Authors:** George Turabelidze, Jay E. Gee, Alex R. Hoffmaster, Farrin Manian, Cindy Butler, David Byrd, Stephanie Schildknecht, Lina Chavez Hauser, Mary Duncan, Rhonda Ferrett, Dana Evans, Crystal Talley

**Affiliations:** Missouri Department of Health and Senior Services, Jefferson City, Missouri, USA (G. Turabelidze, C. Butler, D. Byrd, S. Schildknecht, L. Chavez Hauser, C. Talley);; Centers for Disease Control and Prevention, Atlanta, Georgia, USA (J.E. Gee, A.R. Hoffmaster;; Mercy Hospital, St Louis, Missouri (F. Manian, M. Duncan, R. Ferrett, D. Evans)

**Keywords:** *Bacillus cereus*, bacteria, infant, newborn, neonate, cluster, colonization, disinfection, air flow sensor, ventilator

## Abstract

We investigated *Bacillus cereus*–positive tracheal aspirates from infants on ventilators in a neonatal intensive care unit. Multilocus sequence typing determined a genetic match between strains isolated from samples from a case-patient and from the air flow sensor in the ventilator. Changing the sterilization method for sensors to steam autoclaving stopped transmission.

Because of ubiquity in the environment, the recovery of *Bacillus* species from clinical specimens is often considered a clinically inconsequential contamination. Nevertheless, an accumulating body of literature suggests that contamination with this organism should not be routinely dismissed (*1*). Severe and lethal *Bacillus cereus* infections have been described in newborn infants, with higher frequency among premature infants. The types of *B. cereus* infections in newborns included central nervous system, respiratory tract, primary bacteremia, and sepsis (*2*–*4*). Nosocomial outbreaks of *B. cereus* implicating hospital linens, manual ventilation balloons, contaminated diapers, and contaminated ventilator equipment have also been reported (*5*–*9*).

## The Study

The Missouri Department of Health and Senior Services conducted this investigation in response to the hospital’s identification of an increased number of tracheal aspirates that were positive for *B. cereus* collected from newborns who were on ventilators during March–May, 2011. All tracheal aspirate culture results obtained in the Neonatal Intensive Care Unit (NICU) during January 2010–June 2011 were reviewed. NICU data was also searched for positive *B. cereus* culture from other specimens, such as blood, body fluids, or tissues. Investigators thoroughly evaluated respiratory management practices in the unit by direct observation, respiratory records review, and an interview with the respiratory therapist.

Several environmental cultures were obtained from the flow sensors of the unit’s ventilators over the 1-month period. *B. cereus* isolates were forwarded to the Centers for Disease Control and Prevention to be molecularly characterized by using multilocus sequence typing (MLST) (*10*). DNA was prepared from bacterial cultures as described (*11*). The DNA was used as a template in PCRs with the primers described on the *Bacillus cereus* MLST Web site (www.pubmlst.org/bcereus) for the 7 loci which define the MLST scheme. The sequences for the loci *glpF*, *gmk*, *ilvD*, *pta*, *pur*, *pycA,* and *tpi* were then assigned allele designations. The combination of the 7 alleles determines a given sequence type. A greater number of alleles that match between strains indicates a higher level of relatedness (*10*). Prevalence of *B. cereus–*positive specimens was compared by using the Mann-Whitney U test.

**Figure 1:**
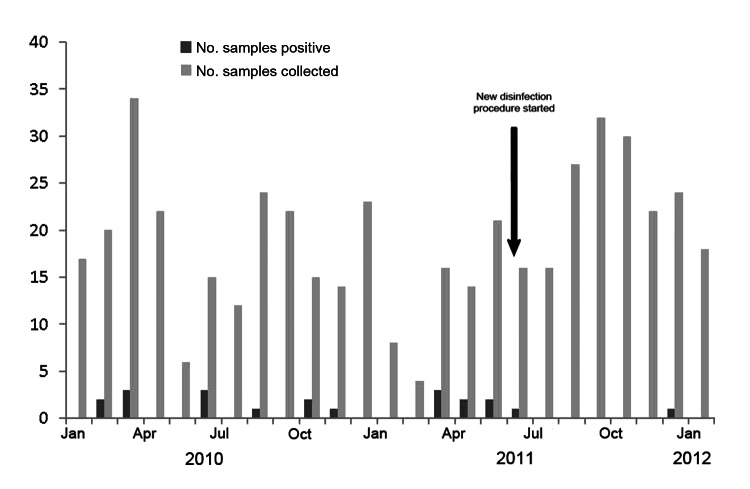
Epidemiologic curve of *Bacillus* spp.–positive tracheal aspirates from newborns on ventilators, January 2010–January 2012.

Retrospective analysis of tracheal aspirate culture results showed significant increase (p = 0.039) in *B. cereus* isolation between March and May, 2011 (Figure 1). No *Bacillus* spp. were isolated from blood, other body fluids, or tissues during the study period. The chart review of the case-patients comprising the cluster of *B. cereus* colonization revealed that none received a diagnosis of clinical *B. cereus* infection. All patients were treated with vancomycin or tobramycin, or both, for indications not related to *B. cereus* in tracheal aspirate. One case-patient died 108 days later without evidence that *B. cereus* contributed to the outcome. All other case-patients recovered and were discharged.

**Figure 2:**
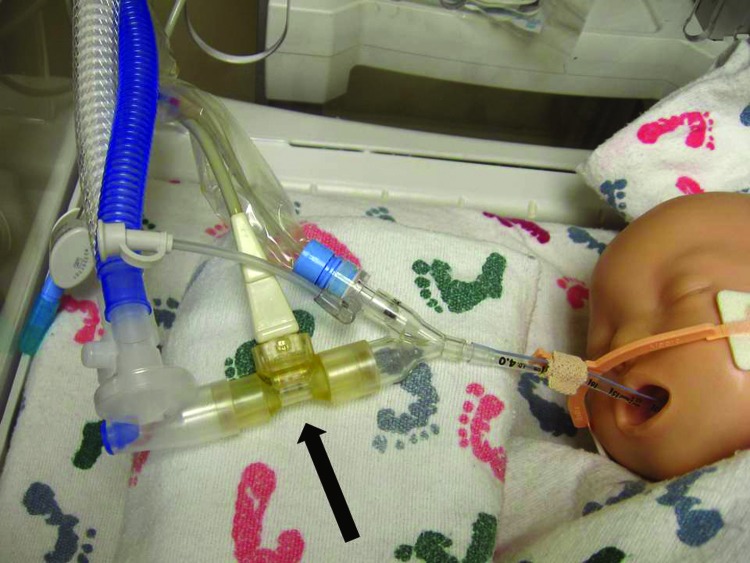
Draeger Evita v500 respirator. Arrow indicates Neoflow air flow sensor.

Investigation of the ventilation procedures in the NICU revealed that most equipment used for respiratory care was disposable, designated for single-patient use. The Draeger Evita v500 ventilator (Draeger Medical Inc., Telford, PA, USA; www.draeger.us/sites/enus_us/pages/hospital/evita-xl.aspx) was used for mechanical ventilation of infants who were intubated to treat severe respiratory compromise. The Draeger Evita V500 is a microprocessor controlled ventilator offering both mandatory and spontaneous ventilation modes for adult, pediatric, and neonatal patients. Heated and humidified gas flows from the ventilator unit, through the inspiratory circuit and NeoFlow air flow sensor to the patient through an endotracheal tube. Upon exhalation, gas flows back through the air flow sensor into the expiratory circuit and returns to the ventilator through the expiratory flow sensor and exhalation valve. In addition to the ventilator, reusable respiratory equipment comprised a proximal air flow sensor, expiratory flow sensor, exhalation valve, and circuit temperature probe. The sensor closest to the newborn’s mouth was an air flow sensor located inside the disposable ventilation circuit (Figure 2). From 9 environmental cultures obtained from 9 air flow sensors, 1 was positive for *Bacillus* spp., and was later confirmed as *B. cereus* by the State Public Health Laboratory.

MLST was performed for 8 *B. cereus* isolates from case-patients and for 1 environmental isolate from the air flow sensor. We were able to fully characterize 4 of the 9 isolates (Table). One locus for the remaining 5 strains did not yield an amplicon for sequencing after repeated attempts and, thus, could not be assigned a sequence type. The isolates that included sequence type (ST) 73 and ST94 were closely related to each other because they differed by merely 1 locus, *gmk*. The strains that were not fully typed because of the inability to obtain sequences for locus *pta* were also closely related to ST73 or ST94 because the other loci matched. There was 1 match between strains isolated from 1 case-patient and the air flow sensor, which was ST73. The contaminated air flow sensor was then sterilized by using a steam autoclave. A repeat culture of this sensor after sterilization was negative.

**Table:** Alleles and sequence types determined for *B. cereus* isolates associated with contaminated ventilator air flow sensor linked to colonization of newborns

Isolate origin	Alleles							Sequence type*
	*glpF*	*gmk*	*ilvD*	*pta*	*pur*	*pycA*	*tpi*	
Patient 1	13	29	9	Null	9	12	31	ND
Patient 1	13	8	9	Null	9	12	31	ND
Patient 2	13	29	9	14	9	12	31	94
Patient 3	13	8	9	14	9	12	31	73
Patient 4	13	29	9	14	9	12	31	94
Patient 5	13	29	9	Null	9	12	31	ND
Patient 6	13	8	9	Null	9	12	31	ND
Patient 7	13	29	9	Null	9	12	31	ND
Airflow sensor	13	8	9	14	9	12	31	73
*ND, not determined.								

We found that air flow sensors were routinely disinfected by placing them in a container with 70% alcohol solution for 60 minutes. After discovery of the air flow sensor contaminated with *B. cereus*, the disinfection policy was changed. All air flow sensors were first soaked in Enzol enzymatic detergent (ASP, Irvine, CA, USA; www.aspjj.com/us/products/enzol) solution and then sent for steam autoclave sterilization at 134°C (273.2°F). After implementation of new disinfection and sterilization procedures, no new cases of *B. cereus* tracheal colonization were identified in the nursery. In this cluster, contaminated proximal air flow sensors were the likely source of tracheal colonization with *B. cereus* in newborn infants, supported by a genetic match by MLST between a strain isolated from 1 case-patient and the contaminated air flow sensor.

## Conclusions

*B. cereus* transmission from contaminated respiratory equipment has been reported in other geographic areas. In the Netherlands, an outbreak of *B. cereus* infections in a pediatric intensive care unit caused by contaminated reusable ventilator air flow sensors was described (*7*). Switching to disposable air flow sensors stopped colonization with *B. cereus* in that unit. In Canada, an outbreak of *B. cereus* infections among patients in an adult ICU was linked to colonized ventilator circuitry (*8*). In the United Kingdom, reusable ventilator circuits were also identified as the cause of a *B. cereus* outbreak among intubated NICU patients (*9*).

Our investigation underscores the necessity of close monitoring of occurrences of *Bacillus* spp. in tracheal aspirates since clustering of such cases could be an indication of single source contamination. In our investigation, *B. cereus* isolates were either ST73, ST94, or closely related to those sequence types. ST73 and ST94 are associated with strains previously described as having caused illness in elderly persons. Strains with ST73 were implicated in cases of septicemia (*12*), and of sepsis and pneumonia (*13*). Strains with ST 94 were recovered from patients with pneumonia (*14*). *B. cereus* strains harboring *B. anthracis* plasmids such as pXO1, have also been associated with severe and fatal respiratory infections (*15*).

All case-patients in our investigation were considered to be colonized with *B. cereus* without clinical implications. Since all of them received intravenous antimicrobial drugs effective against *B. cereus*, it is conceivable that the clinical course of those patients could have been different without such treatment.

*Bacillus* spp. in tracheal aspirate cultures should not be routinely viewed as clinically insignificant and further testing to determine exact strain should be considered under appropriate clinical and epidemiologic circumstances. Proper disinfection of the entire ventilator circuit as recommended by the equipment manufacturer is crucial in avoiding potentially lethal *B. cereus* infections.
